# Supplementary material for: Infectious Inflammatory Processes and the Role of Bioactive Agent Released from Imino-Chitosan Derivatives Experimental and Theoretical Aspects
Source: Polymers (Basel). 2022 Apr 30;14(9):1848. doi: 10.3390/polym14091848 (PMC9100066; doi:10.3390/polym14091848)
Supplement: Supplementary file 1 [file polymers-14-01848-s001.zip › polymers-1622911-supplementary.pdf]

Supporting Information for

Infectious Inflammatory Processes and the Role of Bioactive Agent Released from Imino-Chitosan Derivatives Experimental and Theoretical Aspects

Loredana Himiniuc [1], Razvan Socolov [2], Vlad Ghizdovat [3], Maricel Agop [4,5,*], Emil Anton [2], Bogdan Toma [1],
Lacramioara Ochiuz [6], Decebal Vasincu [7], Ovidiu Popa [8] and Viviana Onofrei [9]

1 "Grigore T. Popa" University of Medicine and Pharmacy Iasi, 700115 Iasi, Romania; loredana.himiniuc@umfiasi.ro (L.H.); bogdan.toma@umfiasi.ro (B.T.)
2 Department of Obstetrics and Gynecology, "Grigore T. Popa" University of Medicine and Pharmacy Iasi, 700115 Iasi, Romania; razvan.socolov@umfiasi.ro (R.S.); emil.anton@umfiasi.ro (E.A.)
3 Department of Biophysics and Medical Physics, "Grigore T. Popa" University of Medicine and Pharmacy Iasi, 700115 Iasi, Romania; vlad.ghizdovat@umfiasi.ro
4 Department of Physics, "Gheorghe Asachi" Technical University of Iasi, 700050 Iasi, Romania
5 Academy of Romanian Scientists, 050094 Bucharest, Romania
6 Department of Pharmaceutical and Biotechnological Drug Industry, "Grigore T. Popa" University of Medicine and Pharmacy, 700115 Iasi, Romania; lacramioara.ochiuz@umfiasi.ro
7 Department of Dental and Oro-Maxillo-Facial Surgery, "Grigore T. Popa" University of Medicine and
Pharmacy, 700115 Iasi, Romania; decebal.vasincu@umfiasi.ro
8 Department of Emergency Medicine, "Grigore T. Popa" University of Medicine and Pharmacy, 700115 Iasi, Romania; ovidiu.popa@umfiasi.ro
9 Department of Internal Medicine (Cardiology), "Grigore T. Popa" University of Medicine and Pharmacy, 700115 Iasi, Romania; viviana.aursulesei@umfiasi.ro
* Correspondence: magop@tuiasi.ro

Citation: Himiniuc, L.; Socolov, R.; Ghizdovat, V.; Agop, M.; Anton, E.; Toma, B.; Ochiuz, L.; Vasincu, D.; Popa, O.; Onofrei, V. Infectious Inflammatory Processes and the Role of Bioactive Agent Released from Imino-Chitosan Derivatives Experimental and Theoretical Aspects. *Polymers* **2022**, *14*, x. https://doi.org/10.3390/xxxxx

Academic Editor(s): Andrea Sorrentino

Received: 17 February 2022
Accepted: 21 April 2022
Published: date

Publisher's Note: MDPI stays neutral with regard to jurisdictional claims in published maps and institutional affiliations.

Copyright: © 2022 by the authors. Submitted for possible open access publication under the terms and conditions of the Creative Commons Attribution (CC BY) license (https://creativecommons.org/licenses/by/4.0/).

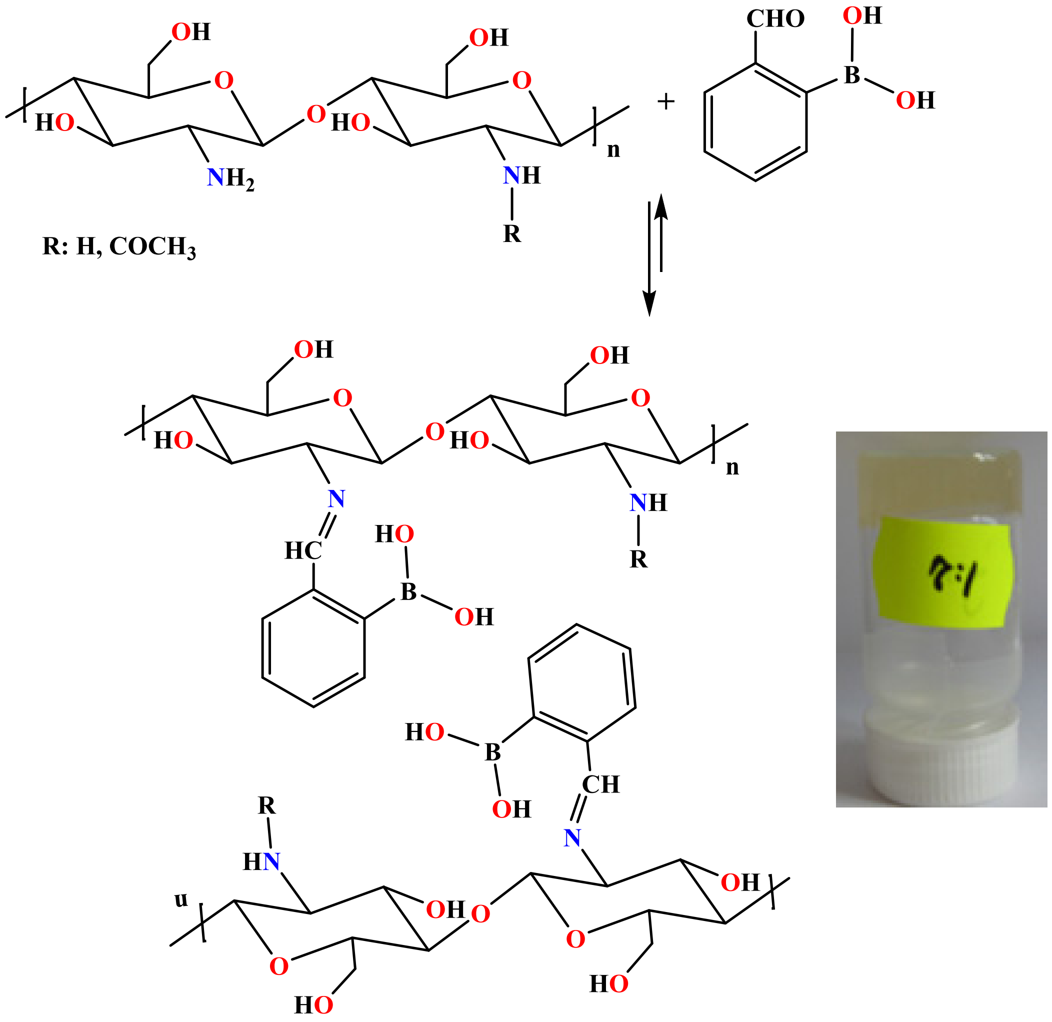

Figure S1. Schematic representation of the synthesis of studied hydrogels, highlighting the imine units and the organization of the imine-chitosan chains to form the hydrogel.

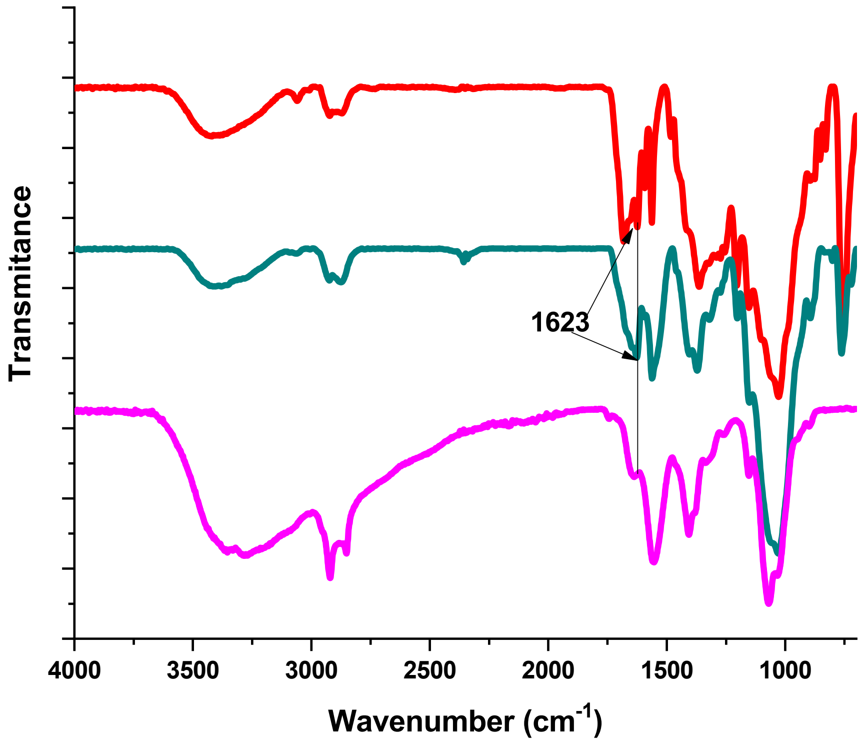

Figure S2. FTIR spectra of chitosan (magenta), **H0.071** (cyan) and **H0.142** (red), proving the occurrence of imine units in hydrogels by characteristic vibration band at 1623 cm^{-1}.
